# Hermit crabs perceive the extent of their virtual bodies

**DOI:** 10.1098/rsbl.2012.0085

**Published:** 2012-02-29

**Authors:** Kohei Sonoda, Akira Asakura, Mai Minoura, Robert W. Elwood, Yukio-P. Gunji

**Affiliations:** 1 Department of Earth and Planetary Sciences, Kobe University, Rokkodai-cho 1-1, Nada, Kobe 657-8501, Japan; 2 Department of Biology, Kobe University, Rokkodai-cho 1-1, Nada, Kobe 657-8501, Japan; 3 School of Biological Sciences, Queen's University Belfast, Belfast BT7 1NN, Northern Ireland, UK

**Keywords:** body, hermit crab, tool use, adaptation, part and whole

## Abstract

A flexible body image is required by animals if they are to adapt to body changes and move effectively within a structurally complex environment. Here, we show that terrestrial hermit crabs, *Coenobita rugosus*, which frequently change shells, can modify walking behaviour, dependent on the shape of the shell. Hermit crabs walked along a corridor that had alternating left and right corners; if it was narrow at the corner, crabs rotated their bodies to avoid the wall, indicating an awareness of environmental obstacles. This rotation increased when a plastic plate was attached to the shell. We suggest that the shell, when extended by the plate, becomes assimilated to the hermit crab's own body. While there are cases of a tool being assimilated with the body, our result is the first example of the habitat where an animal lives and/or carries being part of a virtual body.

## 1. Introduction

Animals must adapt and control their changing bodies, e.g. when a deer re-grows antlers. The phenomenon has been investigated in humans with the rubber hand illusion and in animals by examining how they use tools as extensions of their body [1]. In both cases, new parts are accepted as being their own and under their own control [2]. Using tools (or other constructions [3]) as extensions of the body may have consequences for perception and cognition, as the object becomes embedded as an integrated functional body component, a process known as embodiment [2,4,5].

This may be exemplified by learning to drive a car. A driver is assumed to assimilate his/her own specific position with that of the whole car. Even without a crash that potentially reveals the true dimensions, the driver gains knowledge of the length and width of the car. Full comprehension of the car body (whole body) occurs dynamically when steering the car between obstacles.

Here, we replace the driver and car by a hermit crab, *Coenobita rugosus*, and its gastropod shell. Hermit crabs often change between types of shells in terms of size, weight, specific weight and shape [6–8]; some marine species also attach sea anemones to their shells [9]. The hermit crab must then adjust to each new shell and/or anemone. This adjustment provides a simple model for investigating the emergent relationship between a new component and whole body image in non-human subjects. Presumably, animals can adapt, i.e. they have a flexible body image whether the body change is due to growth (e.g. deer antlers), to carrying a large object, or to using tools [1]. Controlling the new part of the body must account for the new weight and balance and the whole body image must change when animals perceive their new size/shape. We test this here by attaching a plastic extension to the shells of terrestrial hermit crabs and assessing their ability to manoeuvre in confined spaces.

## 2. Material and methods

**Figure 1.**
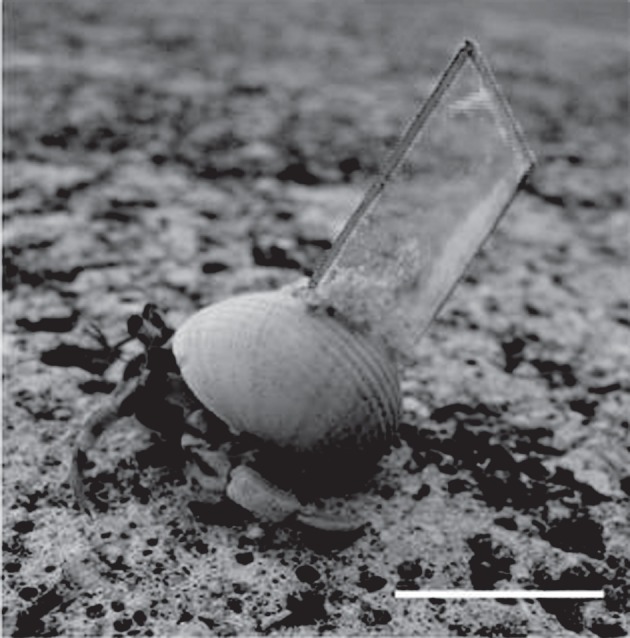
A crab with a plate. Scale bar, 10 mm.

Small *C. rugosus*, in *Ritena plicata* shells (major axis length of 15–20 mm.), were collected from Iriomote Island (Uehara, Taketomi-cho, Yaeyama-gun, Okinawa), in October 2010 at a rate of about 10 per day and kept in plastic containers, fed ad libitum on pineapple and isolated at 27°C for 24 h. We attached plastic plates to some shells with instant glue mixed with sand (figure 1). Experiments were recorded on video (HDC-HS9, Panasonic). The camera was placed horizontally 1000 mm above the centre of the course. Turning angle data were acquired using avidemux2 and the GIMP toolbox.

**Figure 2.**
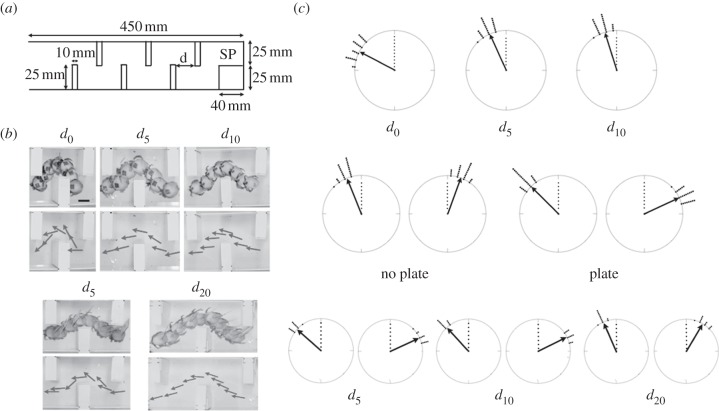
Experimental setting, turning behaviour of crabs and data of turning angles (*a*) plan view of corridor with starting point (SP). (*b*) Crabs moved from right to left through the partitions without (above) and with (below) the extension. Arrows represent crabs' body axis orientations. (*c*) Turning angles of the crabs are given relative to the direction of the centreline of the corridor (dot line in the circle, 0°). Clockwise and anti-clockwise rotations were mirror-images on a 0–180° axis and were superimposed. Arrows represents mean turning angle and the lines on the exterior of the circle indicate data spread. Angles when no plate was present (top) did not differ with rotational direction and are arbitrarily shown as left of centreline but did differ with the plate (bottom). The mean turning angles of *d*_5_ and *d*_10_ (left and right turning correspond to left and right side, respectively) with and without the extension plate (middle).

The corridor was, 450 mm long, 50 mm wide and 40 mm high (figure 2*a*) and had six partitions, placed on the right and left side alternately so as to yield six corners. The variable distance between partitions is expressed by *d_*α*_* such that *d_*α*_* = *L* + *α*, where *L* is the length of the major axis of the crab's shell, and *α* was 0, 5, 10 or 20 mm. Even at *d*_0,_ a crab could pass around the corner. In experiment 1, with natural shells, the interval conditions were *d*_0_, *d*_5_ and *d*_10_. In experiment 2, with plastic plates, the interval conditions were *d*_5_, *d*_10_ and *d*_20_. The plate was 15 mm square, 0.5 mm thick and fixed at 45° right rear angle and 45° elevation angle (figure 1). The crabs in each experiment were then allowed to walk the corridor.

Crabs of both sexes were used (*n* = 72: 40 males; 32 females). Sex was balanced between groups but sex did not affect behaviour in the experiments. Twelve crabs were used in each condition (independent test), without pre-training. We measured the maximum angle between the centreline of the corridor and the direction of crab while turning a corner (turning angle), irrespective of left or right turns (figure 2). A 10° angle during a left turn was recoded as the same as a 10° angle during a right turn. No datum point was below zero. We did not use data from the first and last corner because these differed from the others. Further, we did not use cases in which crabs touched the external walls with body parts such as antennae or legs (not shells or plastic plates) before or after turning. Each crab moved freely until it had turned each corner. We counted only the angle of the first turning in the suitable manner for each rotational direction (left and right turning) for each crab (repeated measure). If the crab stopped walking, it was touched by tweezers to make it withdraw in the shell and was restarted at the starting point. If the crab did not restart, the trial was cancelled.

The angular data were transformed via circular statistics and means compared using the maximum-likelihood method, using a circular normal distribution and analyses of variance for repeated measurements (left and right turn); other factors were independent RM-ANOVA). We use an alpha level 0.05 and used Tukey's HSD post-hoc comparisons to explore significant main factors. Means and variance are given in numerical values in electronic supplementary material.

## 3. Results

In experiment 1, without plates, there was no significant effect of rotational direction (*F*_1,33_ = 0.004, n.s.) or of the interaction between interval distance and rotational direction (*F*_2,33_ = 1.45, n.s.). However, the turning angle declined as the distance (*d*) between the partitions increased (*F*_2,33_ = 149, *p* < 0.0001). Note that the data (figure 2*c*-top) are the means of left and right turns and illustrate deviation from straight on. Each distance (*d*_0_, *d*_5_, *d*_10_) was statistically different from the other two (HSD comparison; *p* < 0.05; figure 2*c*-top).

In experiment 2, crabs were held in a plastic container for 10 min between plate attachment and test. A third of the crabs seemed unbalanced for several steps and attempted to climb the container wall, scrawling their legs in the air. Another third reached up the wall and fell over. The final third managed to walk soon after the attachment. All crabs could walk and balance the shell within 10 min. The interaction term between interval distance and rotational direction was significant (*F*_2,33_ = 4.77, *p* < 0.02; figure 2*c*-bottom). Thus, we analysed that interaction. Turning angles were greater at right than at left turns for all distances (*d*_5_: *F*_1,33_ = 32.5, *p* < 0.0001; *d*_10_: *F*_1,33_ = 51.4, *p* < 0.0001; *d*_20_: *F*_1,33_ = 8.10, *p* < 0.01; figure 2*c*-bottom). There was an effect of distance on left (*F*_2,66_ = 35.8, *p* < 0.0001), and right turns (*F*_2,66_ = 73.7, *p* < 0.0001; figure 2*c*-bottom). With the HSD post-hoc comparisons, we observed that turning angles in *d*_20_ were significantly smaller than in *d*_5_ or *d*_10_ at both left and right turning (HSD comparison; *p* < 0.05; figure 2*c*-bottom).

To examine the effect of the plate, we compared *d*_5_ and *d*_10_ for the two experiments with a three factor RM-ANOVA for circular data. The first-order interaction between attachment and rotational directions was significant (Three-way ANOVA for circular data; *F*_1,44_ = 57.5, *p* < 0.0001; figure 2*c*-middle). Without plates (flat) rotational direction had no effect (*F*_1,44_ = 3.16, n.s.; figure 2*c*-middle), however, in the plate condition crabs turning to the right had a greater turning angle than when turning left (*F*_1,44_ = 86.3, *p* < 0.0001; figure 2*c*-middle). Importantly, crabs with a plate attached turned at a greater angle than those without a plate when turning left (*F*_1,88_ = 112, *p* < 0.0001) and right (*F*_1,88_ = 431, *p* < 0.0001; figure 2*c*-middle). The other first- and second-order interactions were not significant.

## 4. Discussion

Without a plate, direction change was larger in *d*_0_ than *d*_5_ or *d*_10_ (figure 2*b,c*-top), showing that crabs increased rotation of the body and shell to avoid contact with the partition when the interval was narrow. With the plate, direction change was much larger than without (figure 2*b*,*c*-middle). When the plates were connected, crabs did not walk well and most seemed unable to balance but after several seconds they managed to control and balance the shell. Owing to the asymmetry of the plate attachment (figure 1), during right turns the plate sometimes touched the partitions and that might have caused the body rotation. However, the plate never touched the partitions during left turns even though the crab still rotated. During these left turns, the crab appeared to use vision to detect the width of the interval compared with the width of the shell as extended by the plate, but during a right turn it appeared to use touch to detect the width. In addition, in condition *d*_20_, the crabs' turning angles were smaller than those observed in *d*_5_ and *d*_10_ (figure 2*b*,*c*-bottom), showing that the rotation of the body was not due to the plate *per se* but a combination of the plate and specific external stimuli. That is, the increased turning angle with narrow gaps and with the plate attached resulted from assessment of the nature of the environmental obstacle and embodiment of the shell extension.

In this study, we noted that the addition of a plate unbalanced the shell and, initially, most crabs could not walk. However, these crabs quickly managed to balance the shell presumably by altering their leg positions and posture within the shell. However, it is important that the crab should begin to manoeuvre effectively while moving within a complex environment, because touching potential obstructions is likely to increase energy use owing to friction and the jolt may be noxious. However, the ability to avoid partitions is quickly established and this is similar to embodiment noted in monkey and human studies [3,10]. We suggest that embodiment is initiated when crabs first attempt to balance the shell, particularly while walking and when they touched the partitions with the plate when turning right. Subsequent awareness of the extension is shown by the increased turning when partitions requiring either right or left turns were encountered despite the finding that the crabs never touched with the extension in the latter case.

Hermit crabs depend on empty gastropod shells and gather information about shells before they inhabit them [6]. They compare their own shell with that of opponents in shell fights [11] and trade-off avoidance of noxious stimuli with the requirement to keep a preferred shell type [12]. They can even place an anemone in an appropriate position to help balance a shell [9]. That is, these invertebrates are capable of integrating information from different sources to make complex decisions and we have now shown that they rapidly adapt to a changing environment by embodiment.
